# Supplementary material for: Ligilactobacillus murinus Strains Isolated from Mice Intestinal Tract: Molecular Characterization and Antagonistic Activity against Food-Borne Pathogens
Source: Microorganisms. 2023 Apr 4;11(4):942. doi: 10.3390/microorganisms11040942 (PMC10141155; doi:10.3390/microorganisms11040942)
Supplement: Supplementary file 1 [file microorganisms-11-00942-s001.zip › microorganisms-2284854-supplementary.pdf]

Supplementary Materials

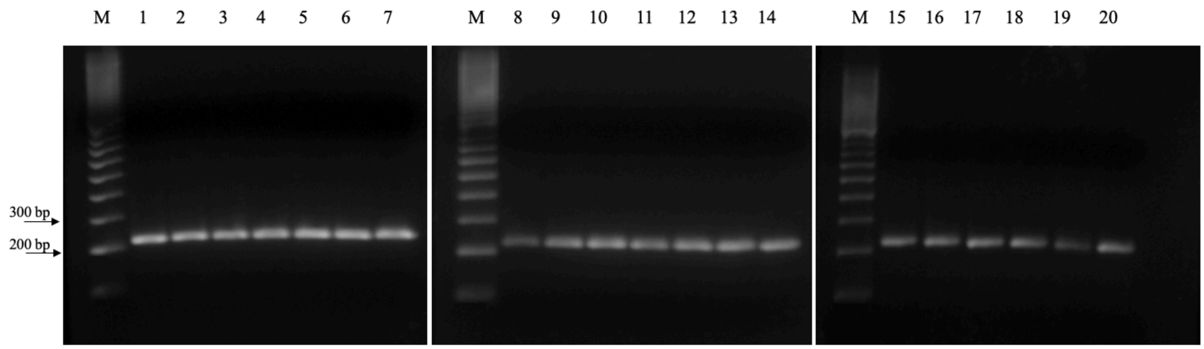

Figure S1. Products obtained through PCR with *Ligilactobacillus animalis* primers for the 18 LAB strains isolated A1, A2, B1, C1, D1, E1, E2, E3, E4, E5, F1, F2, F3, G1, H1, H2, H3 and I1 (lanes 1-18), *Lactococcus lactis* (line 19) and *Lacticaseibacillus rhamnosus* controls (lane 20), GeneRuler™ DNA Ladder Mix as standard of molecular weight (M).

Table S1. Molecular identification (BLAST-NCBI) of isolated LAB strains from mice intestinal tract.

**Strain**	**Description**	**Query Cover**	**E value**	**Per Ident**	**GenBank Accession**
A1	*Ligilactobacillus murinus* strain 700 16S rRNA gene	98%	3e-98	99.51%	MT585453.1
A2	*Ligilactobacillus murinus* strain 700 16S rRNA gene	98%	4e-97	99.02%	MT585453.1
B1	*Ligilactobacillus murinus* strain 700 16S rRNA gene	99%	8e-99	99.51%	MT585453.1
C1	*Ligilactobacillus murinus* strain 700 16S rRNA gene	99%	8e-99	99.51%	MT585453.1
**D1**	***Ligilactobacillus murinus* strain 700 16S rRNA gene**	**99%**	**2e-100**	**100%**	**MT585453.1**
E1	*Ligilactobacillus murinus* strain 700 16S rRNA gene	99%	2e-99	99.51%	MT585453.1
**E2**	***Ligilactobacillus murinus* strain 700 16S rRNA gene**	**99%**	**5e-101**	**100%**	**MT585453.1**
E3	*Ligilactobacillus murinus* strain 700 16S rRNA gene	99%	8e-99	99.51%	MT585453.1
E4	*Streptococcus salivarius* strain1957 16S rRNA gene	99%	1e-91	97.54%	MT597770.1
E5	*Ligilactobacillus murinus* strain 700 16S rRNA gene	99%	2e-94	98.07%	MT585453.1
F1	*Ligilactobacillus murinus* strain 700 16S rRNA gene	100%	8e-99	99.51%	MT585453.1
F2	*Ligilactobacillus murinus* strain 700 16S rRNA gene	99%	1e-96	99.02%	MT585453.1
F3	*Ligilactobacillus murinus* strain 700 16S rRNA gene	94%	6E-85	97.89%	MT585453.1
**G1**	***Ligilactobacillus murinus* strain 700 16S rRNA gene**	**100%**	**8e-99**	**100%**	**MT585453.1**
H1	*Ligilactobacillus murinus* strain 700 16S rRNA gene	99%	3E-98	99.51%	MT585453.1
H2	*Ligilactobacillus animalis* strain 8372 16S rRNA gene	99%	4e-82	95.10%	MT5389999.1
H3	*Ligilactobacillus murinus* strain 700 16S rRNA gene	98%	3e-98	99.51%	MT585453.1
I1	*Ligilactobacillus murinus* strain 700 16S rRNA gene	99%	4e-97	99.02%	MT585453.1
